# Supplementary material for: The interactome of intact mitochondria by cross-linking mass spectrometry provides evidence for coexisting respiratory supercomplexes
Source: Mol Cell Proteomics. 2017 Dec 8;17(2):216–32. doi: 10.1074/mcp.RA117.000470 (PMC5795388; doi:10.1074/mcp.RA117.000470)
Supplement: Supplemental Data [file supp_RA117.000470_133922_0_supp_23978_fzffwf.zip › spectra_annotation/mito_spectra_annotation/187-1-10-1-8-1.pdf]

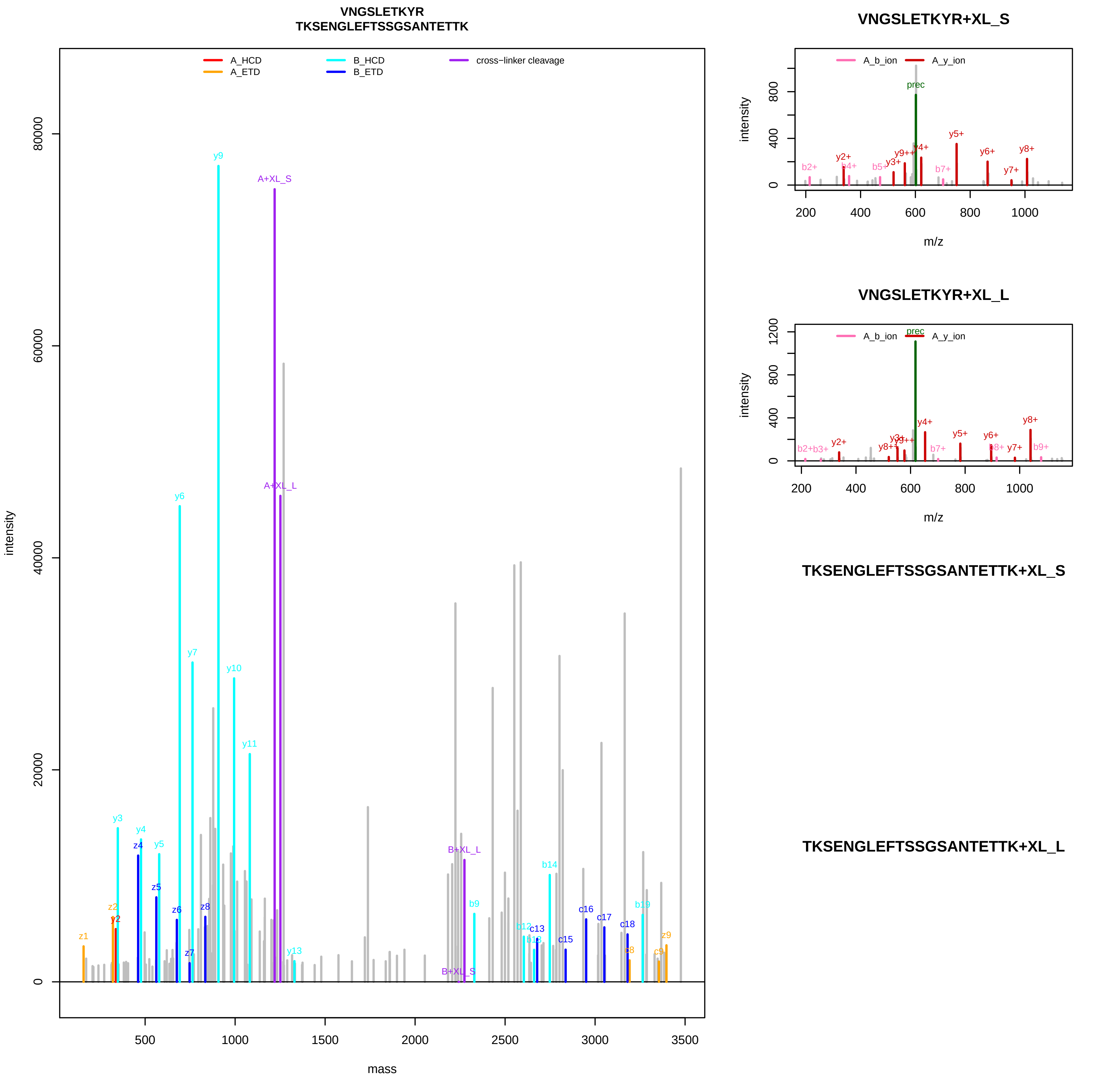
VNGSLETKYR
TKSENGLEFTSSGSANTETTK
A_HCD
A_ETD
B_HCD
B_ETD
cross−linker cleavage
intensity
mass
y9
A+XL_S
A+XL_L
y6
y7
y10
y11
y3
y4
y5
z1
z2
y2
z4
z5
z6
z7
z8
y13
B+XL_L
B+XL_S
b9
b12
b13
c13
c15
b14
c16
c17
c18
c8
c9
b19
z9
VNGSLETKYR+XL_S
A_b_ion
A_y_ion
prec
m/z
VNGSLETKYR+XL_L
TKSENGLEFTSSGSANTETTK+XL_S
TKSENGLEFTSSGSANTETTK+XL_L
